# Effects of dietary arginine supplementation in pregnant mares on maternal metabolism, placental structure and function and foal growth

**DOI:** 10.1038/s41598-019-42941-0

**Published:** 2019-04-23

**Authors:** Morgane Robles, Anne Couturier-Tarrade, Emilie Derisoud, Audrey Geeverding, Cedric Dubois, Michele Dahirel, Josiane Aioun, Audrey Prezelin, Juliane Calvez, Christophe Richard, Laurence Wimel, Pascale Chavatte-Palmer

**Affiliations:** 1 UMR BDR, INRA, ENVA, Université Paris Saclay, 78350 Jouy en Josas, France; 2 IFCE, Station Expérimentale de la Valade, 19370 Chamberet, France; 3 UMR PNCA, AgroParisTech, INRA, Université Paris Saclay, 75005 Paris, France

**Keywords:** Intrauterine growth, Homeostasis

## Abstract

Foals born to primiparous mares are lighter and less mature than those born to multiparous dams. Factors driving this difference are not totally understood. Using 7 multiparous and 6 primiparous standardbred mares, we demonstrated that, in late gestation, primiparous mares were less insulin resistant compared to multiparous mares, and that their foals had reduced plasma amino-acid concentrations at birth compared to foals born to multiparous mares. Vascular development, as observed through structure and gene expression, and global DNA methylation were also reduced in primiparous placentas. Another group of 8 primiparous mares was orally supplemented with L-arginine (100 g/day, 210d to term). L-arginine improved pregnancy-induced insulin resistance and increased maternal L-arginine and L-ornithine plasma concentrations but foal plasma amino acid concentrations were not affected at birth. At birth, foal weight and placental biometry, structure, ultra-structure and DNA methylation were not modified. Placental expression of genes involved in glucose and fatty acid transfers was increased. In conclusion, maternal insulin resistance in response to pregnancy and placental function are reduced in primiparous pregnancies. Late-gestation L-arginine supplementation may help primiparous mares to metabolically adapt to pregnancy and improve placental function. More work is needed to confirm these effects and ascertain optimal treatment conditions.

## Introduction

Modifications of the nutritional environment during early life can affect offspring health at adulthood. This concept is known as Developmental Origins of Health and Disease (DOHaD) and has been first demonstrated in human populations and animal experimental models^1^. It is now well confirmed that this concept also applies to horses^2–4^.

Effects of maternal environment on foal birthweight have rarely been observed and were only reported when the maternal environment was strongly affected (*e.g*. severe undernutrition, embryo transfer into mares of smaller size)^5–7^. The only other factor that has been consistently shown to affect foal birthweight is maternal parity, with primiparous dams producing smaller and/or lighter foals than multiparous dams^8–11^. Foals born to primiparous mares were also shown to remain smaller until 18 months of age, with immature carbohydrate metabolism and reproductive function until 12 months of age^10^. These results were associated with a decreased placental nutrient transfer capacity in primiparous dams, with surface and volume of placental structures, uterine blood flow, as well as gene expression of placental growth factors, growth factor receptors and angiogenic and vasculogenic factors being reduced in primiparous compared to multiparous pregnancies^8–10,12,13^. Nevertheless, placental nutrient transfer capacity *per se* may not be the only factor affecting foetal growth. In all species, maternal insulin resistance is a physiological adaptation to pregnancy to enhance foetal glucose supply^14^. In species such as humans and mice, insulin resistance in response to pregnancy is more pronounced in multiparous compared to primiparous dams, possibly due to an increased body mass index^15–17^. Primiparous dams may then be less able to adapt metabolically to pregnancy.

L-arginine is a conditional essential amino acid in the horse. The particular abundance of arginine in horse milk indicates that L-arginine might be needed in much larger proportions in the foals than in other species^18^. L-arginine is a precursor of ornithine, urea, nitric oxide (NO), polyamines and creatine^19^. NO is the main vasodilatory agent in mammals and is known to be a major player of placental angiogenesis^20^. Indeed, in pregnant mares, L-arginine supplementation in late gestation has been shown to increase uterine artery blood flow^21^. NO and polyamines are also essential for cellular proliferation and differentiation. In the pig, oral L-arginine supplementation during gestation increased the weight of piglets and placentas at birth^22–24^. Moreover, long-term supplementation with L-arginine has been shown to reduce glucose and insulin plasma concentrations following an oral glucose tolerance test in humans^25,26^. L-arginine supplementation could thus increase birthweight and improve maternal metabolism during pregnancy.

The few studies conducted in horses did not demonstrate any effect of L-arginine supplementation on foal birthweight but the possible interaction between L-arginine supplementation and maternal parity was not examined^27–29^.

The two aims of this study were:

- To compare the carbohydrate and amino acid metabolism and further explore differences in placental structure, ultrastructure and function between primiparous and multiparous pregnant mares,
- To study the effects of L-arginine supplementation in primiparous pregnant mares on maternal metabolism, placental structure, ultrastructure and function and on foal birthweight and post-natal growth.

## Results

### Nutrition

**Figure 1:**
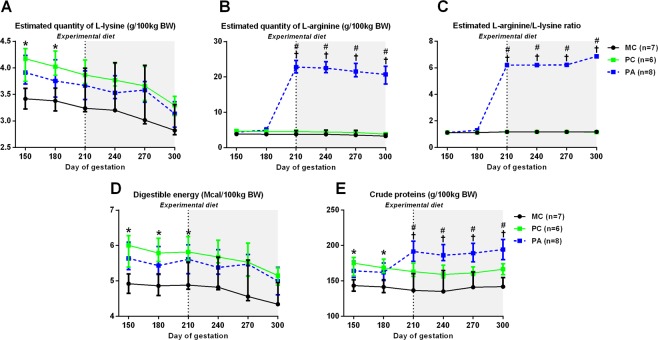
Daily nutritional intake (median and IQR) in broodmares in late pregnancy: estimated L-Lysine (**A**), estimated L-arginine (**B**), estimated L-arginine/L-lysine ratio **(C**), digestible energy (**D**) and crude proteins (**E**) (median and IQR). The experimental diet was administered from 210 days of gestation until foaling, as indicated by the vertical dotted line. MC: Multiparous Control, PC: Primiparous Control, PA: Primiparous Arginine. BW = body weight. ^†^ indicates a statistically significant difference between PA and MC groups (p < 0.05). * indicates a statistically significant difference between PC and MC groups (p < 0.05). ^#^ indicates a statistically significant difference between PC and PA groups (p < 0.05).

Figure 1 summarizes the nutritional protocol.

### Lysine, arginine and proteins

#### Effect of parity

There was a group effect (p = 0.02) for estimated quantities of lysine intake, with MC mares receiving less lysine according to body weight than PC mares at 150 (−22%, p = 0.049) and 180 (−19%, p = 0.049) days of gestation. There was an effect of group (p < 0.0001), time (p < 0.0001) and of the group:time interaction (p < 0.0001) for estimated quantities of arginine intake.

There was an effect of group (p < 0.0001), time (p < 0.0001) and of the group:time interaction (p < 0.0001) for quantities of crude proteins ingested. PC mares received more proteins than MC mares at 150 (−22%, p = 0.04) and 180 (−19%, p = 0.03) days of gestation according to body weight.

#### Effect of arginine treatment

As expected, PA mares received more arginine than MC (+600%, p < 0.0001) and PC (+500%, p < 0.0001) mares from 210 days of gestation according to body weight. The results were the same for the arginine/lysine ratio.

From the 210^th^ day of gestation, PA mares received more protein than MC and PC mares (respectively, +40%, +17%, p < 0.0001, p < 0.0001) according to body weight.

### Energy

#### Effect of parity

There was an effect of group (p = 0.03) and time (p < 0.0001) for digestible energy intake. MC mares ingested less energy than PC mares at 150 (−22%, p = 0.03), 180 (−19%, p = 0.02) and 210 (−19%, p = 0.04) days of gestation according to body weight.

#### Effect of arginine treatment

There was no difference between PA and PC mares for digestible energy intake.

***Summary***: *In summary, primiparous mares received about 20% more lysine, crude proteins and energy than multiparous mares according to body weight from 150 until 180 or 210 days of gestation. L-arginine supplemented mares received more L-arginine and crude proteins compared to non-supplemented mares from the 210*^*th*^ *day of gestation*.

## Maternal measurements

### Body weight

There was a group effect (p < 0.01) and a time effect (p < 0.0001) for body weight of the mares during pregnancy. All primiparous mares (PC and PA) were lighter than MC mares (p = 0.01, p = 0.04, respectively) throughout pregnancy. When corrected with mares’ withers’ height (p < 0.0001), in addition to the other cofactors, no group effect was observed any more. All mares gained a median 59.3 [34.7–65.8] kg during pregnancy and there was no difference between PA and PC mares.

### Body condition score

There was no effect of group nor of the group:time interaction on Body Condition Score (BCS) during pregnancy. From insemination until the beginning of the experimental period, all mares were slightly overweight (median BCS: 3.75 [3.25–4.25]). They lost a median 0.5 [0.25–0.75] BCS during the experimental period and foaled with an optimal body condition score of 3.5 [3.0–4.0].

#### Summary

*In summary, primiparous mares were lighter than multiparous mares during all gestation, but this difference disappeared when wither’s height was taken into consideration in the statistical model. As a result, the body condition score was not different between groups during all gestation*.

## Amino acid plasma concentrations

### Before the experimental diet

Before the beginning of the experimental period, among the 20 amino acids measured, only basal plasma concentrations of cysteine (p = 0.01) and phenylalanine (p = 0.05) differed between groups.

Plasma cysteine (p = 0.02) was reduced in Primiparous mares (PC and PA) compared to MC mares (1.5 [1.3–1.8] pmol/µL and 1.5 [1.1–1.8] pmol/µL *vs* 2.1 [1.8–2.6] pmol/µL, respectively). Phenylalanine concentrations were reduced in the PA *vs* the MC group (21.6 [20.4–23.2] pmol/µL *vs* 32.3 [28.1–35.7] pmol/µL, respectively, p = 0.04) but there was no difference for phenylalanine concentrations between PC and MC mares and no difference between PC and PA mares.

### During the experimental diet

#### Post prandial amino acid concentrations

At 262 days of gestation *i.e*, 52 days after the beginning of L-arginine supplementation, basal data (sampled 16 h after the last concentrated meal) were normalized over the pre-experimental measures to analyze the differences observed between groups.

**Effect of parity**: There was no difference between PC and MC mares for post-prandial amino acid concentrations.

**Figure 2:**
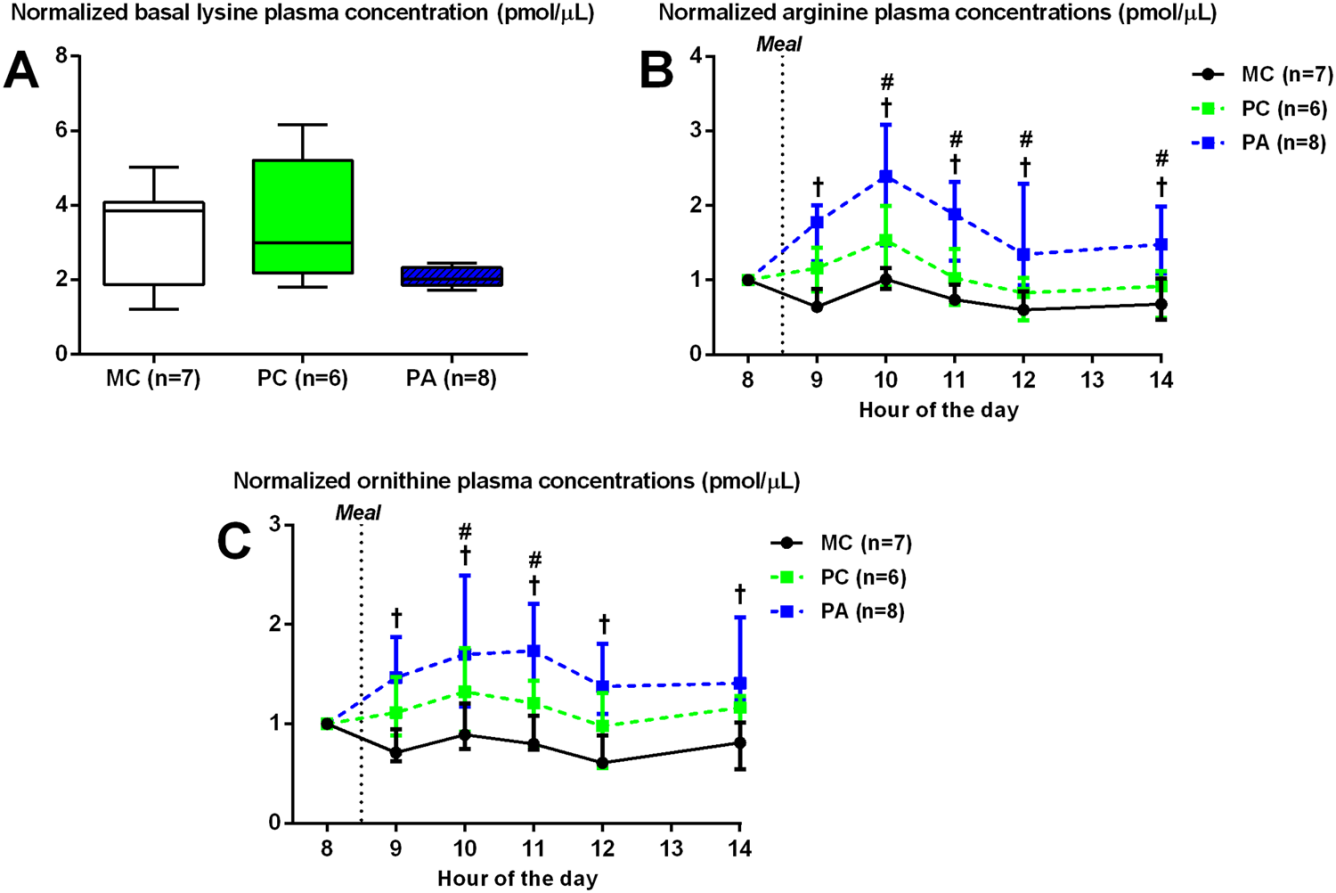
Basal and post-prandial normalized plasma lysine (**A**), arginine and ornithine (**B**) concentrations at 262 days of gestation (median and IQR). (**A**) Basal plasma lysine concentration normalized with pre-experimental period measurements. Group F-test p-value < 0.05. (**B**,**C**) Post-prandial plasma arginine and ornithine concentrations normalized with basal measurements. MC: Multiparous Control, PC: Primiparous Control, PA: Primiparous Arginine. ^†^ indicates a statistically significant difference between PA and MC groups (p < 0.05). * indicates a statistically significant difference between PC and MC groups (p < 0.05).

**Effect of arginine treatment**: There was a group effect for plasma lysine concentrations (p = 0.045) with PA mares tending to have decreased plasma lysine concentrations compared to MC (p = 0.08) and PC (p = 0.07) mares. During the day, after normalizing the data with the basal concentrations of the same day, there was a group effect for plasma arginine (p < 0.0001) and ornithine (p < 0.001) concentrations. PA mares had increased plasma arginine (x2 to 2.5, p < 0.01, p < 0.01, respectively) and ornithine (x1.5, p < 0.01, p = 0.04, respectively) concentrations compared to MC and PC mares up to 6 hours after the meal (Fig. 2). There was no statistical effect for other amino acids.

#### At foaling

**Figure 3:**
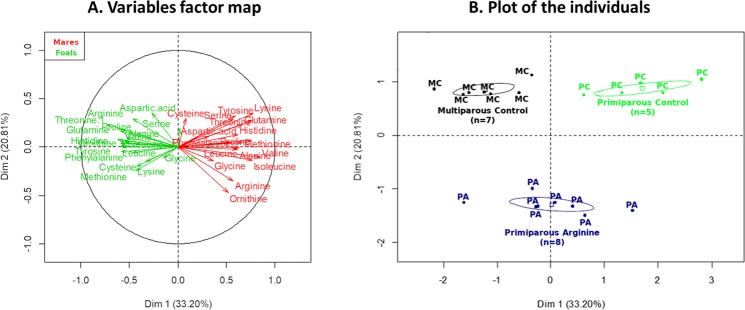
Variables factor map (**A**) and plot of individuals (**B**) of the Multiple Factor Analysis (MFA) applied on mares and foals’ amino acid concentrations at birth. On the left, the variable factor map (**A**) presents the projection of the variables on the two first dimensions. Plasma amino acid concentrations observed in mares are represented in red, and those obtained in foals are represented in green. On the right, the plot of the individuals (**B**) represents the position of the animals on the two first dimensions with mares and foals being considered as pairs. In summary, at foaling, PC mares had higher amino acid plasma concentrations compared to MC mares whereas foals born to PC mares had lower amino acids plasma concentrations than foals born to MC mares. Moreover, PA mares had higher ornithine, but not arginine, plasma concentrations compared to Control mares. MC (black): Multiparous Control (n = 7), PC (green): Primiparous Control (n = 5) PA (blue): Primiparous Arginine (n = 8).

Figure 3 shows the global differences in amino acid plasma concentrations at foaling in mares and foals. Figure 3A shows the relation between the plasma amino acid concentrations in mares (red) and foals (green) and the first two dimensions of the Multiple Factor Analysis. Figure 3B shows the coordinates of the mare-foal pairs according to their experimental group and in relation to the first two dimensions of the Multiple Factor Analysis. The first-dimension accounts for 33.2% while the second-dimension accounts for 20.81% of the total inertia of the dataset. These results mean that 54.01% of the dataset total variability is explained by the two first dimensions.

**Effect of parity**: On the variables factor map, maternal plasma amino acid concentrations are inversely correlated to that of foals (Fig. 3A). Plasma amino acid concentrations in mares (valine, lysine, isoleucine, threonine, proline, glutamine, methionine, leucine, histidine, alanine, tyrosine, arginine, serine, phenylalanine and ornithine, p < 0.05 for each amino acid) are positively correlated to the first dimension whereas foal plasma amino acid concentrations (valine, isoleucine, threonine, proline, glutamine, leucine, histidine, alanine, tyrosine, arginine, phenylalanine and ornithine, p < 0.05 for each amino acid) are negatively correlated to the first dimension. This dimension discriminates between the MC group (p < 0.01, negative correlation) and the PC group (p < 0.001, positive correlation).

**Effect of arginine treatment**: L-ornithine plasma concentrations in mares are negatively correlated to the second dimension (p = 0.03). This dimension discriminates between the PA group (p < 0.0001, negative correlation) and the PC and MC groups (p < 0.0001, p < 0.0001, respectively, positive correlation).

***Summary***: *In summary, L-arginine supplementation roughly doubled plasma arginine concentrations and increased ornithine concentrations approximately by 50% after meals. Moreover, at foaling, primiparous mares had higher amino acid plasma concentrations than multiparous mares whereas foals born to primiparous mares had lower amino acids plasma concentrations than foals born to multiparous mares. Finally, primiparous mares supplemented with L-arginine in late-gestation had higher ornithine, but not arginine, plasma concentrations compared to Control mares. There was no difference in amino acids plasma concentration at birth between foals born to mares supplemented with arginine or not*.

## Maternal metabolism

### Basal glucose, insulin and urea concentrations during gestation

#### Effect of parity

There was a group effect for whole blood basal glucose concentrations (p = 0.02). PC mares had reduced whole blood basal glucose concentrations compared to MC mares at 150 days of gestation (p < 0.01). This difference disappeared afterwards. There was no difference in basal plasma insulin nor urea concentrations between primiparous and multiparous mares at any time.

#### Effect of arginine treatment

There was no difference in basal plasma insulin nor urea concentrations between the three groups during gestation.

Taking into consideration the body weight of the mares did not change these results.

### Glucose metabolism before the experimental diet

There was no difference in glucose metabolism (AIRg, SI, Sg, DI, basal whole blood glucose concentration, basal plasma insulin concentration) before the experimental period between the three groups. Results are shown in Table 1.

**Table 1:** Results of Frequently Sampled Intravenous Glucose Tolerance Test before (median gestational age 142 days) and at the end (280 days of gestation) of the experimental period (median [quartile 1-quartile 3]).

		AIRg	SI	DI	Sg	Basal whole blood glucose concentration (mg/dL)	Basal plasma insulin concentration (mUI/L)
**Before the experimental period**	**MC Group « Multiparous Control » (n** = **6)**	116.1 [80.1–147.2]	7.1 [4.5–7.4]	623.9 [470.5–799.7]	0.017 [0.014–0.024]	79.0 [73.9–83.9]	14.1 [8.5–14.8]
	**PC Group « Primiparous Control » (n** = **6)**	171.4 [150.2–187.8]	5.0 [4.3–5.7]	781.1 [475.6–868.8]	0.015 [0.007–0.020]	66.3 [64.6–84.6]	13.1 [9.9–15.9]
	**PA Group « Primiparous Arginine » (n** = **8)**	116.3 [68.6–149.7]	3.7 [3.1–8.5]	516.2 [329.3–696.1]	0.020 [0.015–0.023]	76.3 [71.1–82.2]	11.8 [9.7–15.4]
**End of experimental period**	**MC Group « Multiparous Control » (n** = **7)**	109.8 [50.3–167.9]	1.6 [1.0–2.6]	**154.6** ^**a**^ [**136.8–205.2]**	0.011 [0.010–0.015]	91.0 [78.0–97.3]	12.7 [8.3–15.6]
	**PC Group « Primiparous Control » (n** = **6)**	150.4 [110.0–205.4]	2.1 [2.0–2.6]	**429.9** ^**b**^ [**251.2–519.1]**	0.014 [0.010–0.017]	93.0 [78.8–99.6]	14.9 [11.3–21.5]
	**PA Group « Primiparous Arginine » (n** = **8)**	114.5 [73.0–131.4]	1.6 [1.1–4.2]	**244.2** ^**a**^ [**143.6–264.1]**	0.009 [0.007–0.015]	86.6 [80.5–90.6]	9.3 [6.3–12.2]

AIRg = Insulin response to glucose.

SI = Insulin sensitivity.

DI = Disposition index (AIRg * SI).

Sg = Glucose effectiveness.

Different superscript letters indicate a p < 0.05 between groups.

### Glucose metabolism at 280 days of gestation

Whether or not the first trimester FSIGT analyses were included in the statistical model did not change the results.

#### Effect of parity

There was no effect of group on AIRg, SI, Sg, whole blood basal glucose and plasma insulin concentrations. The disposition index (multiplication of SI and AIRg), however, was increased in PC compared to MC (2.8-fold, p = 0.01) (Table 1).

#### Effect of arginine treatment

There was no effect of group on AIRg, SI, Sg, whole blood basal glucose and plasma insulin concentrations but the disposition index was increased in PC compared to PA (1.8-fold, p = 0.049).

### Glucose, insulin and urea postprandial concentrations

**Figure 4:**
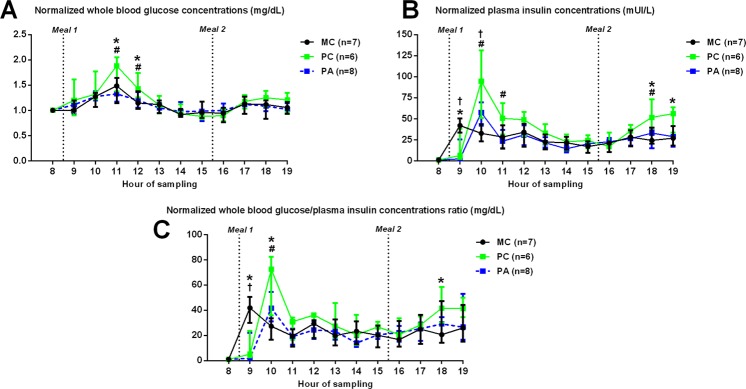
Post-prandial normalized whole blood glucose concentrations (**A**), plasma insulin concentrations (**B**) and whole blood glucose/plasma insulin ratio (**C**) at 262 days of gestation (median and IQR). MC: Multiparous Control, PC: Primiparous Control, PA: Primiparous Arginine. ^†^ indicates a statistically significant difference between PA and MC groups (p < 0.05). * indicates a statistically significant difference between PC and MC groups (p < 0.05). ^#^ indicates a statistically significant difference between PC and PA groups (p < 0.05).

Post-prandial data were normalized over the basal measurements to study only the effects of food ingestion between groups. Results are presented in Fig. 4.

Altogether, there was a group:time effect on whole blood basal glucose concentrations (p < 0.001). There was an effect of group (p = 0.02), time (p < 0.0001) and of the group:time interaction (p < 0.0001) on plasma insulin concentrations. There was also an effect of time (p < 0.0001) and of the group:time interaction (p < 0.0001) on the insulin to glucose ratio. There was no effect of group, time, nor of the interaction group:time on plasma urea concentrations (data not shown). Differences between groups are described below.

#### Effect of parity

PC mares had higher glucose concentrations than MC mares at 3 (p = 0.03) and 4 hours (p = 0.04) after the first meal.

PC mares also tended to have increased insulinemia compared to MC (p = 0.09) for the whole day. When comparisons were made according to the time of the day, insulinemia was lower in PC mares 1 hour after the first meal (p = 0.03) and higher 2 hours after the first meal (p < 0.0001) and 3 and 4 hours after the second meal (p = 0.01 and p = 0.03, respectively) compared to MC mares.

The insulin to glucose ratio was increased in PC mares 1 and 2 hours after the first meal (p < 0.01 and p < 0.0001, respectively) and 2 hours after the second meal (p = 0.049) compared to MC mares.

#### Effect of arginine treatment

PA mares had reduced glucose concentrations compared to PC mares at 3 (p < 0.01) and 4 hours (p = 0.047) after the first meal.

Insulinemia was also lower in PA compared to PC (p = 0.05) mares throughout the day. When comparisons were made according to the time of the day, insulinemia was reduced in PA mares 2 and 3 hours after the first meal (p < 0.001 and p = 0.04, respectively) and 3 hours after the second meal (p = 0.02) compared to PC mares. Moreover, insulinemia was reduced in PA mares 1 hour after the first meal (p < 0.01) and insulinemia was increased 2 hours after the first meal (p = 0.04) compared to MC mares.

The insulin to glucose ratio was reduced in PA mares 2 hours after the first meal (p = 0.049) compared to PC mares. The ratio was also decreased in PA mares 1 hour after the first meal compared to MC mares (p < 0.001).

***Summary***: *Altogether, these results show that glucose and insulin concentrations strongly differed between primiparous and multiparous mares, with multiparous mares being more insulin resistant than primiparous mares. In contrast, arginine treated primiparous mares were very similar to multiparous mares for glucose and insulin metabolism*.

## Placental histology

### Placental stereology

**Figure 5:**
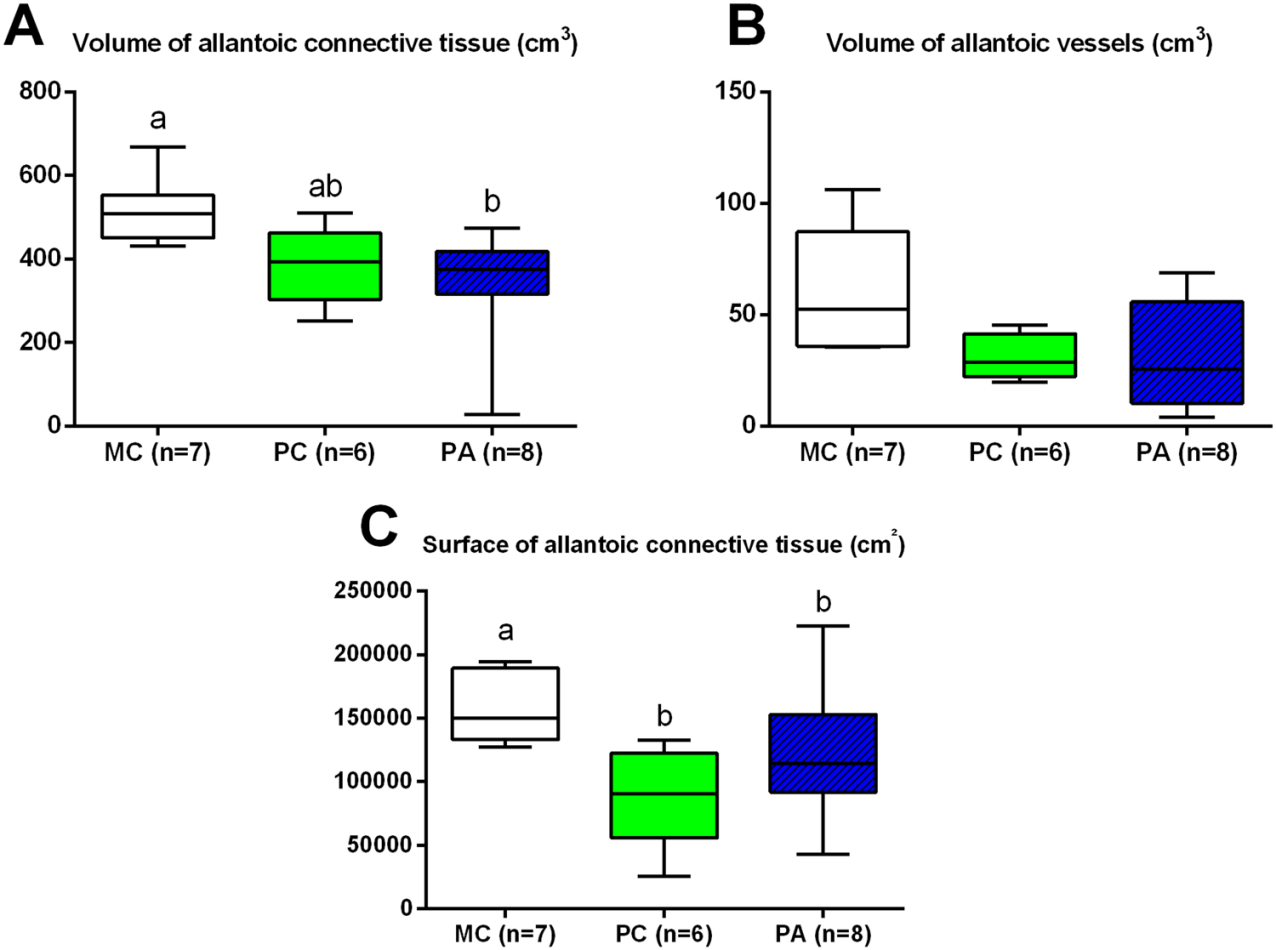
Volume and surface of placental allantoic components at term (median, IQR, min and max). MC: Multiparous Control, PC: Primiparous Control, PA: Primiparous Arginine. Different letters indicate p < 0.05.

Only significant results are presented in Fig. 5. There was a group effect for allantoic connective tissue (p = 0.01) and vessels volume (p = 0.02). There was no effect of the sex of the foal.

#### Effect of parity

The surface of the allantoic connective tissue was increased in MC compared to PC placentas (p = 0.02). MC placentas also had an increased volume of allantoic connective tissue compared to PA placentas (p = 0.03) and tended to have an increased volume of allantoic vessels compared to PA and PC placentas (p = 0.059, p = 0.08, respectively).

#### Effect of arginine treatment

There was no other difference between groups.

### Placental ultrastructure

There was no effect of group nor foal sex on the number of vesicles in the cytoplasm normalized over the surface area, nor on the number of caveolae on the basal lamina normalized over the length of the basal lamina.

## Placental function

### Gene expression

**Figure 6:**
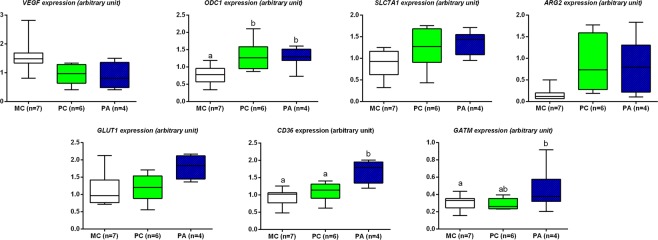
Relative expression of genes involved in vascularization (*VEGF*), nutrient transfer (*SLC7A1*, *ARG2*, *GLUT1*, *CD36*) and arginine metabolism (*OCD1*, *GATM*) in term placentas (median, IQR, min and max). MC: Multiparous Control, PC: Primiparous Control, PA: Primiparous Arginine. Different letters indicate p < 0.05.

Results for genes showing differential expression are presented in Fig. 6. There was no effect of group, nor sex of the foal on *H19*, *IGF1R*, *IGF2*, *IGF2R*, *EGFR*, *TGFβ1 (involved in growth and development)*, *eNOS*, *Flt1*, *KDR (involved in vascularization), SNAT1 (involved in amino-acid transfer) and SRM, SMS* and *GAMT* (involved in arginine metabolism) gene expression. A group effect was observed for *GLUT1* (p = 0.04), *CD36* (p < 0.01), *VEGF* (p = 0.049), *GATM* (p = 0.02) and *ODC1* (p = 0.02).

Altogether, placentas of male foals had increased expression of *GLUT1* (p < 0.01) and *LPL* (p < 0.01) compared to placentas of female foals.

#### Effect of parity

The expression of *VEGF* (p = 0.08, p = 0.08, respectively) tended to be increased and that of *ODC1* (p = 0.03, p = 0.03, respectively), *SLC7A1* (p = 0.06, p = 0.09, respectively) and *ARG2* (p = 0.09, p = 0.08, respectively) was or tended to be decreased in MC compared to PC and PA placentas.

#### Effect of arginine treatment

The expression of *GLUT1* (p = 0.06, p = 0.09, respectively), *CD36* (p < 0.01, p = 0.05, respectively) and *GATM* (p = 0.01, p = 0.09, respectively) was or tended to be increased in PA placentas compared to MC and PC placentas.

### DNA methylation

**Figure 7:**
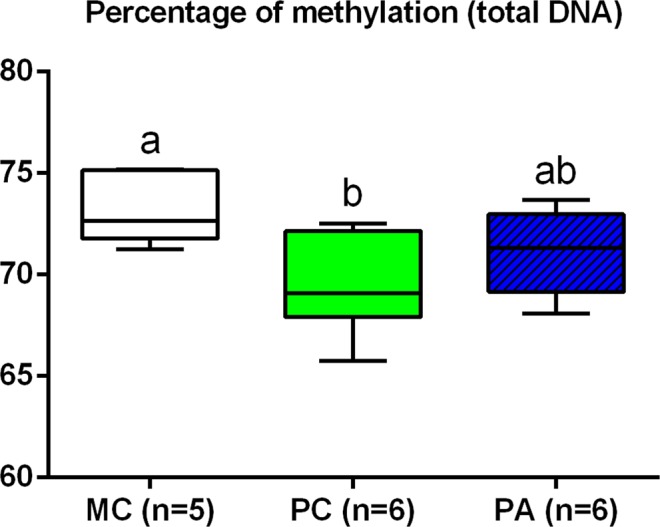
Percentage of methylation in total DNA of term placentas. MC: Multiparous Control, PC: Primiparous Control, PA: Primiparous Arginine. Different letters indicate p < 0.05.

There was a group effect for global DNA methylation (p = 0.03) (Fig. 7).

#### Effect of parity

Global DNA methylation was increased in MC compared to PC term placentas (+3.8%, p = 0.03).

#### Effect of arginine treatment

Global DNA methylation did not differ between MC and PA nor between PC and PA.

***Summary***: *In summary, primiparity was associated to a decreased expression of genes involved in vascularization (VEGF), arginine and ornithine metabolism (ARG2 and OCD1) and arginine and lysine transport (SLC7A1). Global DNA methylation was increased in placentas of multiparous mares*.

*L-arginine supplementation of primiparous mares was associated with an increased expression of genes involved in glucose and fatty acids transport (GLUT1 and CD36) and arginine metabolism (GATM)*.

## Foeto-placental measurements at birth and foals’ growth

### Foeto-placental measurements at birth

**Figure 8:**
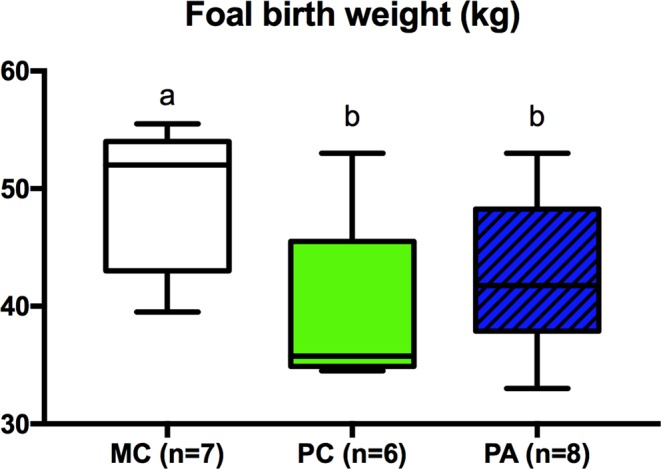
Birth weight of foals (kg) according to group. MC: Multiparous Control, PC: Primiparous Control, PA: Primiparous Arginine. Different letters indicate p < 0.05.

There was no effect of group on placental weight, surface and volume, foal measurements, glycemia nor on colostrum IgG concentrations at birth. Foal birthweight differed between groups (p = 0.04), with MC foals (median: 52 [45.0–53.3] kg) being born heavier than PC (median: 35.5 [35.0–41.3] kg, p = 0.06) and PA (median: 41.7 [38.6–44.8] kg, p = 0.03) foals (Fig. 8).

### Growth of foals

There was no effect of group, nor foal sex on foal wither’s height until 1 year of age. There was no effect of group, nor sex of foal on foals’ body weight until 2 months of age.

#### Summary

*In summary, at birth, multiparous dams produced larger foals compared to primiparous mares, but L-arginine supplementation did not affect foal weight nor foal height*.

## Discussion

The present study aimed to investigate the effects of oral supplementation with L-arginine in late pregnancy on maternal glucose and amino acid metabolism, placental and newborn foal development, taking into consideration mare parity. Metabolic adaptation to pregnancy (increased insulin resistance in response to pregnancy) was impaired in primiparous mares, as well as expression of genes involved in placental transfers. L-arginine supplementation improved the metabolism of primiparous mares to a level close to that of multiparous mares but had only moderate effects on placental function. The following discussion first addresses the differences due to parity, and subsequently the effects of L-arginine supplementation.

In the present study, in late gestation, primiparous mares produced more insulin in response to feeding and had an increased disposition index compared to multiparous mares. At foaling, plasma amino acid concentrations were increased in primiparous mares but that of their foals were decreased compared to multiparous mares and foals. Volume and surface of allantoic components (connective tissue and vessels) were decreased in primiparous mares’ placentas, together with a decreased *VEGF* gene expression and global DNA methylation. Expression of genes involved in L-arginine, L-lysine and L-threonine transfer and degradation were increased in primiparous placentas.

Primiparous mares were smaller and lighter compared to multiparous mares and received an increased amount of feed relative to body weight. Body weight of the mare was included in the statistical model to correct this difference. Although primiparous dams received more digestible energy and crude proteins during late-gestation than multiparous mares, as expressed per kg of body weight, their body weight and body condition score remained parallel to those of multiparous dams, demonstrating that either the difference did not have strong biological effect, either that the needs of primiparous mares were increased compared to those of multiparous mares during gestation. The last hypothesis is unlikely. Indeed, in sows, primiparous dams appear to have decreased energy needs and increased feed efficiency compared to multiparous sows during gestation^30^. Moreover, in a previous study in our laboratory (unpublished data), primiparous dams had increased body condition score gains compared to multiparous dams during pregnancy, while eating the same amount of energy, which correlates well with what has been observed in sows. Finally, differences in nutrient intake could also have affected maternal metabolism. Because primiparous mares received more cereals related to body weight, it could have been expected that both groups of primiparous mares would have an increased glucose and insulin response to feeding. The fact that primiparous arginine supplemented mares differed from primiparous control mares in terms of glucose and insulin responses and that they were not significantly different from multiparous control mares seems to invalidate this hypothesis. Moreover, differences in glucose and insulin responses to meal between primiparous and multiparous control mares are unlikely to be explained only by differences in nutrient ingestion. In a previous study, we compared post-prandial metabolic response between pregnant mares receiving only forage or forage and flattened barley and differences in peak whole blood glucose concentrations were lower (0.59 mg/dL) compared to what was observed in the present study (1.7 mg/dL)^31^. Thus, the difference in energy and proteins intake between primiparous and multiparous mares was unlikely to affect the physiological parameters analysed in the present study.

There was no difference in basal nor post prandial plasma amino acid concentrations between primiparous and multiparous dams during gestation. It is known that pregnant primiparous sows have increased needs in lysine in late-gestation compared to multiparous sows^32^. In sows, the first gestation occurs concomitantly with growth, explaining the difference in lysine needs. In the present study, primiparous mares were 4 to 5 years old at insemination and were thus at the very end of their skeletal growth^33^. They most probably had the same lysine needs compared to older multiparous mares during gestation. Nevertheless, at foaling, increased plasma amino acids concentrations were observed in primiparous mares, associated to decreased amino acid plasma concentrations in their newborn foals, suggesting decreased placental amino acid transfer in primiparous mares.

There was no difference in glucose metabolism between primiparous and multiparous mares at 142 days of gestation. At 280 days of gestation, however, the disposition index (product of insulin sensitivity and pancreas insulin production, indicating the organism’s capacity to dispose of blood glucose into peripheral tissues) was higher in primiparous dams compared to multiparous dams. Moreover, at 247 days of gestation, primiparous mares had an increased glycaemic and especially insulinemic response (as calculated by the insulin/glucose ratio) to feeding compared to multiparous mares. At the beginning of gestation, pregnant mares become more efficient to absorb glucose and produce more insulin in response to the increased blood glucose concentrations, which enhances maternal capacity of glucose storage in peripheral tissues^14^. Later on in gestation, however, insulin sensitivity, glucose tolerance of peripheral tissues, as well as pancreatic production of insulin in response to glucose are reduced to enhance glucose transfer to the growing foeto-placental unit^14,34^. The present results indicate that metabolic adaptations to late-gestation conditions are reduced in primiparous compared to multiparous dams. The increased DI in primiparous mares could affect glucose transfer to the foetus in late gestation and then participate, in addition to the decreased vascularization and surface of exchange structure in placentas, to the observed low birth weight of foals^8–11^.

Increased volume and surface of allantoic connective tissue were observed in multiparous placentas with a tendency for increased allantoic vessels’ volume compared to primiparous placentas. These modifications in placental structure were associated with a tendency for increased *VEGF* gene expression in multiparous compared to primiparous term placentas. Previous works have shown that surface and/or volume of haemotrophic and histotrophic exchange structures are increased in multiparous placentas^8–10^, but this was not observed here. The small sample size and the weak statistical power (<50% for each measured variable) probably accounts for the lack of difference observed for exchange structures between parities.

**Figure 9:**
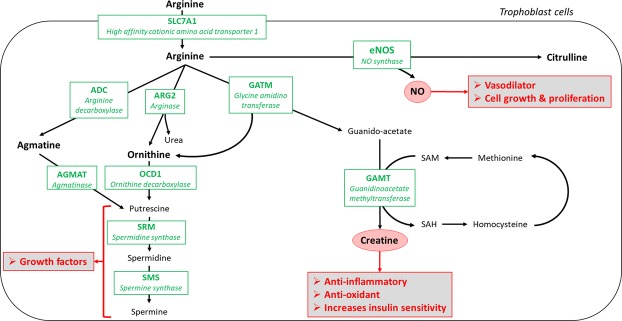
Schematic figure illustrating arginine metabolism in the trophoblastic cell. The genes analysed are indicated in green. Arginine may have beneficial effects through the synthesis of creatine, polyamines (putrescine, spermidine, spermine) and NO as indicated in frames. Through the catabolism of creatine, arginine also plays a role in the methionine metabolism and could possibly affect DNA methylation.

Gene expression of *ARG2*, *ODC1* and *SLC7A1* was increased in primiparous compared to multiparous placentas. Effect of parity on placental gene expression has been scarcely studied in the horse and expression of genes evolved in amino acid transfer (*SLC38A1*, *SLC38A2*) has not been observed to be affected in primiparous placentas^13^. In humans, gene expression of amino acid transporters was shown to be downregulated in placentas of primiparous compared to multiparous mothers^35^. Conversely, in the present paper, the gene expression of *SLC7A1* (involved in cell transport of lysine and arginine) was increased in primiparous placentas and expression of *SLC38A1* (encoding for sodium-coupled neutral amino acid transporter 1) did not differ between groups. ARG2 is involved in the degradation of L-arginine into L-ornithine and OCD1 is involved in the degradation of L-ornithine into putrescine^19^ (Fig. 9). The increased gene expression of the transporter of L-arginine and L-ornithine and of genes involved in their catabolism in primiparous placentas may be related to metabolic placental differences between parities. In humans, the gene expression of glutamate dehydrogenase, involved in the production of α-ketoglutarate from glutamate, is increased in primiparous placentas^35^, suggesting that primiparous dams’ placentas could possibly need more energy than those of multiparous dams. Moreover, the differences in mare and foal plasma amino acids at birth may indicate a more efficient amino acid transfer in multiparous mares. Further analyses exploring protein expression and amino acid transfer capacity are needed to confirm these hypotheses. In addition, modifications in structure and function of the endometrium between primiparous and multiparous mares may also have altered the maternal-foetal amino acid transport. To our knowledge, however, differences in endometrial function in relation to parity are not known in the horse at these stages of pregnancy.

Finally, global DNA methylation was increased in term placentas from multiparous compared to primiparous mares. This result came as a surprise and suggests that there could be a link between the functional and structural differences between placentas of primiparous and multiparous dams and the differences observed in DNA methylation. Although preliminary, these results highlight the importance of placental epigenetic regulations on the development of the offspring. Gene-specific methylation studies need to be carried out in these species to better understand the mechanisms underlying the growth retardation observed in foals born to primiparous dams at birth. It is indeed impossible to correlate global DNA methylation with expression of genes measured in this study as DNA sequences affected by differential methylation are unknown. To our knowledge, this is the first time DNA global methylation is analysed in the horse placenta, and the first time it is compared between placentas from primiparous and multiparous dams, regardless of the species.

There was no difference in placental weight, surface and volume nor in foals’ wither’s height at birth. Foal birthweight was, however, higher in multiparous compared to primiparous groups, which is consistent with what has been observed in previous studies^8–11^. The absence of differences in placental biometry between parities in the present work may be due to a poor statistical power (<50% for each variable measured at birth) because of the small sample size. Absence of difference for foals’ growth may also be a result of the small sample size, which is a main limitation of this work, especially in a species that is not strongly genetically selected for these specific traits.

Primiparous mares supplemented with L-arginine in late gestation received more crude proteins compared to control mares. In late gestation, the basal plasma lysine concentrations were decreased, and post-prandial L-arginine and L-ornithine increased in L-arginine supplemented compared to control mares. The pancreatic response to feeding and the disposition index were decreased in L-arginine supplemented mares and were similar to those observed in multiparous mares. At foaling, L-arginine supplemented mares had increased L-ornithine plasma concentration compared to control mares, but there was no difference in plasma amino-acid concentrations between foals of both groups. Moreover, L-arginine supplementation did not affect the weight of foals at birth and there was no effect of L-arginine supplementation on placental biometry, structure and ultra-structure, nor on global DNA methylation. Finally, there was an increased gene expression of *GLUT1*, *CD36* and *GATM* in placentas of L-arginine supplemented compared to control mares.

One main limit of this work is the fact that both diets were not isonitrogenous. As a result, primiparous mares supplemented with L-arginine received more crude proteins than primiparous control mares. In another study performed in the horse, additional crude protein provided by L-arginine supplementation was normalized in the control group using urea^27^. In that study, L-arginine supplementation reduced uterine fluid accumulation after foaling in mares. This work showed that L-arginine may have effects *per se*, independent of the amount of nitrogen ingested. Moreover, the effects of protein excess in pregnant mares are unknown. In the present case, however, urea plasma concentrations were not different and remained stable between groups during gestation and after meals, indicating that excess protein received by PA mares was likely excreted in urine after degradation. Indeed, in humans, a 90-days 30 g L-arginine supplementation (equivalent to 90 g in a 500 kg horse as calculated using the metabolic body weight (BW^0.75^)) did not modify serum urea concentrations but increased urea urinary excretion^36^.

L-arginine supplemented primiparous mares had decreased basal L-lysine plasma concentrations compared to control primiparous and multiparous mares. Arginine and lysine are known to compete for the same transporters. There was, however, no difference in lysine absorption between control and L-arginine supplemented mares after feeding. Effects of L-arginine supplementation on basal and postprandial lysine plasma concentrations are controversial in other species, but L-arginine supplementation, length of supplementation and L-arginine/L-lysine ratios are different between studies that thus become hardly comparable^36–38^. In the horse, supplementing non-pregnant mares during 1 day with 120 g of L-arginine (compared to 100 g in the present study) led to a decreased post-prandial plasma concentration of lysine, methionine, histidine and proline^28^. The follow-up of postprandial amino-acid concentrations was, however, performed only during one day in that study and progressive adaptations of horses to the diet, such as a putative intestinal adaptation, could not be observed. As a matter of fact, in the pig, intestinal expression of *SLC7A7* and *SLC7A1* genes (involved in lysine and arginine transport) was shown to be increased after 60 days of L-arginine supplementation^39^. Alternatively, the effects on basal lysine concentrations may be due to a decreased lysine renal reuptake. Indeed, increased urine lysine concentrations have been observed after 90 days of 30 g L-arginine supplementation in humans (equivalent to 90 g in a 500 kg horse as calculated using the metabolic body weight (BW^0.75^))^36^.

Post-prandial increases of L-arginine and L-ornithine plasma concentrations in PA mares were expected, as L-arginine is a direct precursor of L-ornithine through the urea cycle^19^. At foaling, PA mares had increased L-ornithine plasma concentration compared to PC mares without changes in foal plasma concentrations. L-ornithine is a precursor of putrescine, a polyamine precursor of other polyamines such as spermine and spermidine^19^, that are involved in growth and development and decreased in intra uterine growth retarded foetuses^40^.

The DI was decreased in L-arginine supplemented primiparous mares compared to control primiparous mares. Decreased insulin production in response to feeding in late-gestation was also observed. Thus, the differences observed between primiparous and multiparous mares for DI and insulin production disappeared with L-arginine supplementation. L-arginine has been shown to have dual effects on pancreas insulin production. In the short term, it increases insulin secretion^41^, but insulin secretion is reduced in the long term^42^. In humans, it has been shown that long-term L-arginine supplementation increases glucose tolerance through a decrease in plasma glucose and insulin concentrations during an oral glucose tolerance test^25,26^. L-arginine could thus help primiparous mares to adapt metabolically to gestation by affecting pancreatic insulin secretion and by increasing glucose tolerance. These metabolic modifications may lead to increased glucose availability for placental transfer. Mechanisms underlying the effects of L-arginine on pancreatic response to increased glycemia in primiparous mares are unknown. Moreover, FSIGT results are difficult to interpret, as no differences in short-term insulin production (AIRg) and insulin sensitivity (SI) were observed, while the disposition index (DI = AIRg*DI) was nonetheless affected.

There was no effect of L-arginine supplementation on placental structure and ultrastructure at term. Increased gene expression of *GLUT-1*, *CD36* and *GATM* was observed in placentas of primiparous mares supplemented with L-arginine. The increased expression of *GLUT-1* may have induced an increased maternal-foetal glucose transfer, in addition or in association to the decreased post-prandial insulin production observed in arginine supplemented primiparous mares. CD36 is a fatty acid transporter. Its expression is increased in placentas of obese ewes^43^ and women^44^. Increased placental gene expression in the present study may affect fatty acid transplacental transfers but also fatty acid use and storage in placentas. GATM is involved in the production of guanido-acetate from L-arginine. Guanido-acetate can be phosphorylated or transformed into creatine, a reaction catalysed by the GAMT enzyme. As *GAMT* gene expression was not increased in PA compared to PC placentas, creatine production may not have been affected by L-arginine supplementation in placentas. Finally, global DNA methylation in PA term placentas was not different from PC and MC placentas. Creatine synthesis from guanido-acetate occurs through the transfer of a methyl group from the S-adenosyl methionine (SAM) methyl donor (Fig. 9). SAM is also involved in methionine metabolism as a methyl donor and its use for creatine synthesis may decrease the methyl pool for DNA methylation. The fact that global DNA methylation in PA placentas was no different compared to PC and MC groups seem to validate the previous hypothesis but is also consistent with the absence of modification of *GAMT* gene expression.

There was no effect of L-arginine supplementation on foal birth weight. Moreover, placental efficiency did not seem to be affected by L-arginine supplementation. Aside from statistical power, it is possible that L-arginine supplementation occurred too late in gestation, long after placental formation^45–48^. The lack of difference in foal birthweight correlated well with the absence of difference in placental biometry at term. In the horse, however, a normal foal birth weight is not predictive of the absence of long-term effects. It is therefore not possible to conclude of an absence of effects of L-arginine maternal supplementation in the development of the offspring.

Finally, the results observed in this study may not be reproducible in other breeds, especially breeds that are known to have strong metabolic differences compared to Saddlebred horses such as ponies, Arabian and Iberian horses^49^, as it has been shown that effects of L-arginine on glucose metabolism were inconsistent depending on ethnic origin in humans^26^. Moreover, interaction effects between diet and L-arginine supplementation on metabolism have been observed in rodents^50^, highlighting the fact that diet composition (high sugar or high fat) may also modify the effects of L-arginine supplementation.

## Conclusion

In conclusion, the difference in foal birthweight between primiparous and multiparous mares may be the result of a decreased placental nutrient transfer due to decreased placental surface of exchange structures, uterine blood flow, but also to reduced placental nutrient transfer function in primiparous pregnancies. Decreased amino acid placental transfer and increased insulin response in primiparous dams may affect the *in-utero* growth and development of foals and explain the prolonged immaturity observed in foals in previous studies.

L-arginine supplementation in late gestation decreased basal lysine and increased post-prandial L-arginine and L-ornithine plasma concentrations in pregnant primiparous mares. L-arginine supplementation affected maternal metabolism, increased the placental expression of genes involved in nutrient transfer and the L-ornithine plasma concentration in newborn foals.

The effects of L-arginine supplementation in late gestation in primiparous mares thus appear to be moderate on placental structure and function and foetal growth and development. In the present study, L-arginine supplementation appeared harmless but long-term effects for both the mare and the foal have not been studied. Moreover, effects of earlier supplementation^51^ or longer supplementation should be explored.

## Material and Methods

### Animals management and arginine supplementation

#### Ethical statement

The animal studies were approved by the local animal care and use committee (“Comité des Utilisateurs de la Station Expérimentale de Chamberet”) and received ethical approval from the local ethics commitee (« Comité Regional d’Ethique pour l’Expérimentation Animale du Limousin ») under protocol number 5-2013-5. All experiments were performed in accordance with the European Union Directive 2010/63/EU.

#### Animals management and experimental diet

Experimental horses were raised in the “Institut Français du Cheval et de l’Equitation” experimental farm (Chamberet, France, 45°34′55.17″N, 1°43′16.29″E, 442 m). All animals were vaccinated and dewormed as for standard care. Twenty-one warmblood mares (French Anglo-Arab and Selle Français breeds) were artificially inseminated with the semen of the same French Anglo-Arab stallion between the 11^th^ of May and the 4^th^ of July. From insemination, all the mares were managed in the same pastures with free access to water and mineral salts (Krouner Rumi, CTH, France). From the 15^th^ of October (mean gestational age 130 ± 18 days), the mares were housed in individual boxes with wheat straw bedding and fed hay, haylage and a homemade mix of flattened barley and mineral and vitamins supplement (Excel-Prima-S, Chauveau Nutrition, France) distributed in two meals (8:30 AM and 4:30 PM). When housed in boxes, the mares had access at least twice a week to a dry lot. From the 210^th^ day of gestation, the mares were allocated to one of three groups:

- A “Multiparous Control” group (MC, n = 7) and a “Primiparous Control” group (PC, n = 6) that were fed, in addition to the previously described diet, pellets composed of barley and cane molasses.
- A “Primiparous arginine” group (PA, n = 8) fed the same pellets as for the control groups but to which 10% of L-arginine had been added. This provided altogether 100 g of L-arginine (Ajinomoto, Japan) per day and per mare.

**Figure 10:**
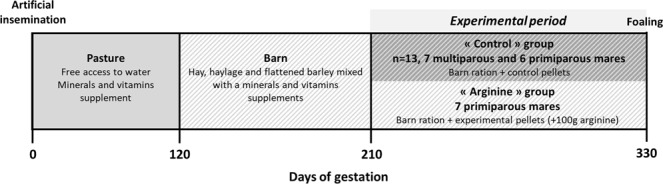
Experimental design from insemination until foaling in pregnant mares. From insemination to the 120^th^ day of gestation, mares were housed as one herd in pasture. Until the 210^th^ day of gestation, they were housed in individual boxes and fed hay, haylage and a home-made mix composed of barley and a minerals and vitamins supplements. From the 210^th^ day of gestation, they were allocated into two groups and fed the barn ration as well as control pellets composed of barley and molasses or experimental pellets composed of barley, molasses and 10% of L-arginine.

The experimental protocol is presented in Fig. 10. A summary of the mares’ age, parity, wither’s height, body weight and body conditions score before the beginning of the experimental period is presented in Table 2.

**Table 2:** Repartition of broodmares between groups “Multiparous Control”, “Primiparous Control” and “Primiparous Arginine” according to age, parity, withers’ height, body weight and body condition score at 210 days of gestation (median [quartile 1-quartile 3]).

	Age (years)	Parity	Withers’ height (cm)	Body weight (kg)	Body condition score (1–5 scale)
**Group « Multiparous Control » (n = 7)**	8 [8–10]^a^	2 [1.5–2]^a^	162 [159–164]^a^	623.7 [603.4–628.1]^a^	4.25 [3.5–4.25]
**Group « Primiparous Control » (n = 6)**	4 [4–4]^b^	0 [0–0]^b^	152.5 [149.8–155.3]^b^	543.0 [506.7–571.4]^b^	3.75 [3.63–4.25]
**Group « Primiparous Arginine » (n = 8)**	4 [4–4]^b^	0 [0–0]^b^	156.5 [152–158.3]^b^	522.4 [494.0–533.5]^b^	4.38 [3.88–4.5]

Different superscript letters indicate a p < 0.05 between groups.

Both diets were calculated to be isoenergetic between groups and to allow the mares to maintain a stable body condition score during all gestation. Because of the L-arginine supplementation, the PA diet was, however, higher in crude proteins, in arginine/proteins and in arginine/lysine ratios. Table 3 presents the nutritional information of feed given to pregnant mares with the amount of feed ingested by the mares and Table 4 the energy, proteins and crude fibres and the lysine, arginine, and arginine/proteins and arginine/lysine ratios as calculated following the NRC requirement system^52^. Individual refusals were weighed each day so as to calculate individuals’ daily intake.

**Table 3:** Daily individual quantities of feeds expressed as kg of brute matter given to pregnant mares of groups “Control” and “Arginine” from the 10^th^ of October (mean gestational day 150) to parturition.

Gestational day	Type of hay	Group « Multiparous Control »				Group « Primiparous Control »				Group « Primiparous Arginine »			
		Hay (kg)	Haylage (kg)	Barley mix (kg)	Control pellets (kg)	Hay (kg)	Haylage (kg)	Barley mix (kg)	Control pellets (kg)	Hay (kg)	Haylage (kg)	Barley mix (kg)	Experimental pellets (kg)
**120–150**	H1	8	3	2.5	0	8	3	2.5	0	8	3	2.5	0
**150–180**	H1	8	3	2.5	0	8	3	2.5	0	8	3	2.5	0
**180–210**	H1	8	3	2.5	0	8	3	2.5	0	8	3	2.5	0
**210–240**	H1	8	3	1.5	1	8	3	1.5	1	8	3	1.5	1
**240–270**	H1	8	3	1.5	1	8	3	1.5	1	8	3	1.5	1
**270–300**	H2	8	3	1.5	1	8	3	1.5	1	8	3	1.5	1
**300–330**	H2	8	3	1.5	1	8	3	1.5	1	8	3	1.5	1

H1 and H2 indicate when a different hay batch was used. Shaded areas indicate the experimental period.

**Table 4:** Nutrient composition of feedstuff given to pregnant mares of groups “Control” and “Arginine” from the 10^th^ of October (mean gestational day 150) to parturition.

	Digestible Energy (Mcal/kg DM)	Crude proteins (g/kg DM)	Lysine (g/kg DM)	Arginine (g/kg DM)	Arginine/lysine	Arginine/crude proteins
**Hay 1**	2.38	58.2	1.2	1.2	1	0.02
**Hay 2**	2.59	71.5	1.2	1.2	1	0.02
**Haylage**	2.51	101.2	1.1	0.5	0.45	0.005
**Barley mix**	3.22	140.8	4.4	6.1	1.4	0.04
**Control pellets**	3.69	102.8	3.5	5.9	1.7	0.06
**Experimental pellets**	3.73	294.5	3.2	108.1	33.8	0.37

Digestible energy was calculated using the NRC equations: for concentrates DE = 4.07–0.055 ADF, for forages DE = 2.118 + 0.01218 CP - 0.000937 ADF - 0.00383 (NDF - ADF) + 0.04718 EE + 0.02035 NFC - 0.0262 Ash (where NFC = 100 - %NDF - %CP - %EE - %Ash). Arginine and lysine contents were assayed for control and experimental pellets and were estimated from the NRC amino acid tables of feedstuff for barley (as barley grain hulless), hay (as grass-legume mixtures predominantly grass, hay, mid-maturity) and haylage (as grass-legume mixtures predominantly grass, silage, mid-maturity).

## Measurements and blood sampling

### Body measurements and blood sampling

All measurements and sampling were performed without analgesia or anaesthesia. Body condition score (INRA 1 to 5 scale^53^) and body weight were measured in mares every two weeks from insemination until parturition. From the 150^th^ day of gestation, basal jugular vein blood samples were collected into 10 ml EDTA-coated tubes each month between 8:15 and 9:00 AM, 16 h after the last meal of concentrates and before the first meal of the day. Blood samples were also collected within 30 min maximum after foaling. Whole blood glucose, was immediately measured using an automated analyser (Freestyle optium, Abbott, USA). Plasma was extracted after 10 min of centrifugation at 3 500 g and samples were stored at −20 °C until plasma insulin and amino acid concentration assays.

The 12^th^ of February (mean gestational age 247 [235–265] days), jugular vein blood samples were collected in pregnant mares each hour over a 11-h period, starting at 8:00 AM and ending at 7:00 PM. Whole blood glucose was immediately measured using an automated analyser (Freestyle optium, Abbott, USA), and plasma was separated after centrifugation and stored at −20 °C until plasma insulin and amino acid concentration assays.

All mares foaled naturally and foaling was supervised. At birth, before suckling, foals were weighed, measured for withers’ height, thoracic perimeter, front leg length, shoulders, hip and cannon width. In total, 11 male and 10 female foals were born. The MC group was composed of 5 male and 2 female foals, the PC group of 3 female and 3 male foals and the PA group of 5 females and 3 male foals. Blood samples were collected for measurement of plasma amino acid and whole blood glucose concentrations. A sample of colostrum was also collected and immunoglobulin G concentration was measured using a refractometer (colotest, IFCE, France).

### Glucose metabolism

#### Frequently sampled intravenous glucose tolerance test

At a median gestational age of 142 [133–155] days of gestation, and at exactly 280 days of gestation, a modified frequently sampled intravenous glucose tolerance tests (FSIGT) was performed in pregnant mares. The FSIGT method calculates quantitative indexes that estimate simultaneously both glucose tolerance and insulin sensitivity^54^. As presented previously^55,56^, animals had free access to hay and water during the test and from the evening before. Both jugular veins were catheterized (Introcan, BBraun, Germany) 30 min before the beginning of the test. One catheter was used for infusion and the other one for sampling. Glucose (0.30 g/kg of body weight) was injected over 5 min and 10 ml samples were collected into EDTA-coated tubes at −5 min and 5, 7, 13 and 19 min after the glucose injection started. At 20 min, a diluted solution (2.51 mUI/L) of insulin (Umuline 100 mUI/L, Eli Lilly, USA) was injected over 1 min at the concentration of 15 mUI/kg of body weight. Blood samples were collected at 5, 15, 25, 40, 70, 100, 130 and 160 min after insulin injection. Whole blood glucose concentration was immediately analysed using a glucometer (Freestyle optium, Abbott, USA). Samples were kept on ice until centrifugation and stored at −20 °C until use.

#### Bergman’s minimal model

FSIGT indexes, such as glucose effectiveness (Sg), acute insulin response to glucose (AIRg), insulin sensitivity (SI) and the disposition index (DI) were calculated using the Bergman’s minimal model^54^ on the MinMod Millennium software (Ver 6.02, MINMOD Inc., 2001)^57^. A schematic representation of the mathematical model has been described previously^55^. As a biological explanation of the Bergman’s model, glucose and insulin are produced and released by the liver and the pancreas respectively in a basal state (basal plasma glucose concentration, Gb; basal plasma insulin concentration, Ib). After a meal, or an intravenous injection of glucose, plasma glucose concentrations increase. From the blood compartment, the glucose will be stored in peripheral tissues by two types of glucose transporters:

- By insulin independent glucose transporters. The glucose efficiency (Sg) index is the capacity of the glucose to suppress endogenous glucose production and to mediate its own disposal independently of insulin.
- By insulin dependent glucose transporters. The elevated concentration of plasma glucose stimulates the production of insulin by the pancreatic β-cells. The acute insulin response index (AIRg) reflects β-cells responsiveness, *i.e*., the production of insulin by the pancreas during the first 10 minutes after glucose injection. Insulin is then transferred into the interstitial space (P3, insulin introduction rate) from the bloodstream and reaches the peripheral tissues to mediate glucose disposal by insulin dependent glucose transporters (X, insulin action). With time, insulin action declines at a rate calculated by the P2 proxy. The Insulin sensitivity (SI) index is then calculated as P2/P3. Finally, the disposition index (DI) (calculated as the product of SI and AIRg) is used to describe the whole-body insulin sensitivity.

### Plasma assays

#### Insulin assays

Plasma insulin concentrations were measured using an AlphaLISA human insulin immunoassay kit (PerkinElmer, USA) as previously described and validated^55^. The minimum level of detection was 5.3 mUI/L. Intra- and inter-assay coefficients of variation were 6% and 7%, respectively.

#### Amino acid assays

Plasma amino acid concentrations (Aspartic acid, serine, glycine, glutamine, histidine, arginine, threonine, alanine, proline, cysteine, tyrosine, valine, methionine, ornithine, lysine, isoleucine, leucine and phenylalanine) were measured using a high-pressure liquid chromatography (HPLC)-coupled fluorescence method. Proteins were precipitated using 20 µL of sulfosalicylic acid (1 g/ml) in 250 µL of plasma, after a 15 min centrifugation at 5976 rpm. Supernatants were then diluted (1:1) and 5 µL of alpha-aminobutyric acid was added as an internal standard (final concentration 125 pmol/µL). L-ornithine was added to the calibration standard at a concentration of 100 pmol/µL because it was not included in the commercial kit. Samples were derivatized using a commercial kit (AccQ fluor reagent kit, Waters SAS, France), according to the manufacturer instructions. HPLC analysis was performed on a Shimadzu Prominence system (Shimadzu, Japan). The injection volume was 5 µL, the column temperature was set to 55 °C, the excitation wavelength was 250 nm and the emission wavelength was 395 nm. The chromatograms were recorded and integrated on the LCsolution software (version 2.1, Shimadzu, Japan).

#### Urea assays

Plasma urea concentrations were measured in mares in duplicate by an enzymatic-colorimetric method with a Cobas Mira-analyser, using commercial kits (Provet urea SL, Kitvia, France) and following the manufacturers’ instructions. Intra- and inter-assay coefficients of variation were 5.9% and 5.9%, respectively.

## Placental analyses

### Biometry and sampling

Immediately after delivery, the placentas (allantochorion) were weighted. A picture of the placentas in “F” configuration under a clear Plexiglas sheet marked with 10 × 10 cm squares was taken. The placental gross surface was subsequently measured on the photographs using the ImageJ® software (National Institute of Health) as previously described^6^. Placental volume was evaluated using a graduated water container, by measuring the volume of water displaced after immersing the placenta into the water.

Placental samples including the chorionic villi and the underlaying allantoic membrane were collected within 30 min of delivery above the umbilical cord insertion and on the pregnant and the non-pregnant horns. Samples (n = 3 for each procedure) were either fixed in 4% formaldehyde for histological analysis (1 cm² samples), in 2.5% glutaraldehyde in 0.2 M cacodylate buffer, pH 7.2 for ultrastructural analysis (3 mm^2^ samples) or snap frozen in liquid nitrogen and stored at −80 °C for functional analysis (3 mm^2^ samples). Ultrastructural and functional analyses were performed on umbilical cord samples only while stereology analyses were performed on samples collected above the umbilical cord insertion and in the pregnant and the non-pregnant horns.

### Placental structure

Placental samples were embedded in paraffin. Sections (7 μm) were stained with haematoxylin/eosin for stereological analysis and scanned using the NanoZoomer Digital Pathology® scanner (Hamamatsu Photonics). Surface densities (Sv) and Volume fractions (Vv) of the different components of the allantochorion, *i.e*., microcotyledons and allantois, were quantified by One stop stereology using the Mercator® software (ExploraNova, France^58^). As previously described^59^, the components measured were part of the microcotyledonary region (haemotrophic trophoblast, microcotyledonary vessels, connective tissue and microcotyledons as the sum of all microcotyledonary components) and of the allantoic region (histotrophic trophoblast and allantoic vessels. The chorionic mesoderm and allantoic connective tissue were merged as allantoic connective tissue).

Vv and Sv were multiplied by the total volume of the placenta to obtain an estimation of the absolute volume (cm^3^) and surface (cm^2^) of the components of the allantochorion.

### Placental ultrastructure

Placental samples collected above the umbilical cord insertion were rinsed in cacodylate buffer 0.1 M with sucrose 0.2 M, then immersed in filtered 0.5% Oolong tea extract, post-fixed in 1% osmium tetroxide (O_2_O_4_) and 1.5% potassium ferrocyanide and dehydrated in alcohol and acetone at 37 °C using a KOS microwave (microwave Histo STATION, Milestone medicals, USA). Samples were embedded in Epon resin (Epon 812, EMS, Delta microscopies, France) at 45 °C.

Placental sections, contrasted with lead citrate, were observed on a Hitachi HT-7700 (Hitachi, Japan) transmission electron microscope (TEM) at a tension of 80 kv.

Images were processed using the ImageJ® software (National Institute of Health). For each sample, 10 vessels were randomly chosen. Randomly chosen endothelial cell areas (2–3/vessel) were analysed by counting the number of vesicles in the cytoplasm and the number of caveolae on the basal lamina. Vesicles and caveolae numbers were quantified according to cytoplasm surface (μm^2^) or basal membrane length (μm), respectively (Supplementary Fig. 1).

### Placental gene expression

Gene-specific primers of genes involved in growth and development (*H19, IGF2, IGF1R, IGF2R, TGFB1* and *EGF-R*), nutrient transfer (*SLC2A1, SLC38A1, SLC38A2, CD36*, and *LPL*), vascularization (*VEGFA, FLT1, KDR* and *eNOS*) and arginine metabolism (*GATM, GAMT, ARG2, ODC1, SRM, SMS* and *SLC7A1*), are shown in Table 5. The role of the enzymes encoded by genes involved in arginine metabolism is illustrated in Fig. 9. Total RNA from placental samples collected next to the umbilical cord insertion was isolated as isolated on silica-based columns, using the RNeasy Plus Mini Kit with effective elimination of genomic DNA, following the manufacturer’s instructions (Qiagen, France). Reverse transcription and real time quantitative PCR were performed as previously described^60^, but qPCR were performed on the StepOne Plus system (ThermoFisher Scientific, USA). Data were analysed using QbasePLUS® (Biogazelle, Belgium). Calibrated normalized relative quantities (CNRQ) were also calculated as previously described^60^. For genes involved in growth and development, nutrient transfer and vascularisation, *GAPDH* (Glyceraldehyde-3-Phosphate Dehydrogenase) and *RPL32* (Ribosomal Protein L32), as selected by geNorm, were used as reference genes. For genes involved in arginine metabolism, *SDHA* (Succinate dehydrogenase complex, subunit A), *HPRT* (Hypoxanthine Phosphoribosyl transferase) and *SCAMP3* were used as reference genes.

**Table 5:** Gene-specific primers and accession number.

	Candidate gene	Forward and reverse primers	Accession number
*Growth and development*	*H19*	F 5′-GGACCCCAAGAACCCTCAAG-3′	NR_027326
		R 5′-GGGACTTGAAGAAGTCCGGG-3′	
	*IGF2*	F 5′-TTTCTTGGCTTTTGCCTCGT-3′	NM_001114539.1
		R 5′-CCTGCTGAAGTAAAAGCCGC-3′	
	*IGF1R*	F 5′-CGAGAAGACCACCATCAACAAC-3′	XM_001489765.2
		R 5′-TGGCAGCACTCGTTGTTCTC-3′	
	*IGF2R*	F 5′-GTCGGCTTGCCAGATGAGAT-3′	XM_001491469.2
		R 5′-TACTGATGGAGACGGCCTCA-3′	
	*TGFβ1*	F 5′-TTGATGTCACCGGAGTCGTG-3′	NM_001081849.1
		R 5′- CCACGCGGAGTGTGTTATCT-3′	
	*EGF-R*	F 5′-GTCTGGAAGTTTGCGGATGC-3′	XM_001497730.2
		R 5′-CTTGGGCCCATTTCTTGCAC-3′	
*Nutrient transfer*	*SLC2A1*	F 5′-TGTGCTCATGACCATCGCC-3′	NM_001163971.1
		R 5′-AAGCCAAAGATGGCCACGAT-3′	
	*SLC38A1*	F 5′- AAATGAACTACCCTCCGCCA -3′	XM_001489402.
		R 5′ ACACAGAGGGAGAATTATGCCAA-3′	
	*SLC38A2*	F 5′-ACAGCTCGAACAGCGACTTCA-3′	NM_001081849.1
		R 5′- TTCTTCCCCAAATTCGATTCA-3′	
	*CD36*	F 5′-GGTCTACGCCGTGTTTGGAG-3′	XM_005609038.1
		R 5′-CCGTGCAGAAGCAGTGGTTA-3′	
	*LPL*	F 5′-AGTTGGGTGCCAAAACTTGTG-3′	XM_001489577.2
		R 5′-GCTTGGTGTACCCCGCAGAC-3′	
*Vascularization*	*VEGFA*	F 5′- TACCTCCACCATGCCAAGTG -3′	NM_001081821.1
		R 5′- GTCTCGATTGGACGGCAGTA -3′	
	*Flt1*	F 5′-AGTGTGAGCGGCTCCCTTATG-3′	XM_003363176.1
		R 5′-ATGCCAAATGCAGATGCTTG-3′	
	*KDR*	F 5′-CAGTGGGCTGATGACCAAGA-3′	XM_001916946.2
		R 5′-TCCACCGAAGATTCCATGCC-3′	
	*eNOS*	F 5′-TTCGGGAGAGTGAGCTGGTA-3′	XM_001504650.3
		R 5′-CAATCCCGCGCATCAAAGAC-3′	
*Arginine metabolism*	*GATM*	F 5′-TTTCCAACCCTGATCGACCA-3′	ENSECAT00000014879.1
		R 5′-TGATCATCTGGGATGACGGG-3′	
	*GAMT*	F 5′-GCTGCTGAGGTCCAAGTACT-3′	ENSECAT00000009900.1
		R 5′-CTCTGTGCGGATGTTCTCAC-3′	
	*ARG2*	F 5′-TACACCGCTCACCACTTCAT-3′	ENSECAT00000017770.1
		R 5′-ATCCTGGGAGTTGTGGTACC-3′	
	*OCD1*	F 5′-GACAGCAGAACCATCGTGAA-3′	ENSECAT00000024431.1
		R 5′-TGCGTAGATAATCCTCTCCGG-3′	
	*SRM*	CAGGATGCCTTCGATGTCAT	ENSECAT00000021064.1
		TTGAGGGCCGTCTTCATGAG	
	*SMS*	GGAGATCGTCTGTGTCCCTT	ENSECAT00000018780.1
		GCACACGTGATTTGGGGATA	
	*SLC7A1*	F 5′-TCCGGTCATCTCTGCTTGAA-3′	ENSECAT00000016547.1
		R 5′-CTGACAGGACACCAGGGATT-3′	
*Reference genes*	*GAPDH*	F 5′-CGATGGTGAAGGTCGGAGTAA-3	NM_001163856.1
		R 5′-TGAAGGGGTCATTGATGGCG-3	
	*RPL32*	F 5′-GGGAGCAATAAGAAAACGAAGC-3′	ENSECAG00000007201
		R 5′-CTTGGAGGAGACATTGTGAGC-3′	
	*SCAMP3*	F 5′-CTGTGCTGGGAATTGTGATG-3′	ENSECAG00000014133
		R 5′-ATTCTTGCTGGGCCTTCTG-3′	
	*SDHA*	F 5′-GAGGAATGGTCTGGAATACTG-3′	ENSECAT00000003106.1
		R 5′-GCCTCTGCTCCATAAATCG-3′	
	*HPRT*	F 5′-AATTATGGACAGGACTGAACGG-3′	ENSECAT00000018496.1
		R 5′-ATAATCCAGCAGGTCAGCAAAG-3′	

### Luminometric DNA methylation assay (LUMA) in placentas

Since arginine metabolism is linked to methionine metabolism and thus possibly to DNA methylation, global placental DNA methylation was analysed by LUMA^61^. Briefly, 500 μg of genomic DNA was cleaved for 4 h 00 at 37 °C, using the isochizomeres HpaII (methylation sensitive) and MspI (non-methylation-sensitive) in two separate reactions and in the presence of EcoRI to standardize for DNA amounts (New England Biolabs, USA). The three enzymes were used at 7.5U in Cutsmart Buffer 1 × (50 mM Potassium Acetate, 20 mM Tris-acetate, 10 mM Magnesium Acetate, 100 μg/ml BSA, pH 7.9). The protruding ends were then used as templates for pyrosequencing^62^ with the Pyromark Q24 device and Pyromark Gold Q96 reagents (Qiagen, France). The luminometric signals produced by either the sequential incorporation of C and G nucleotides (reflecting the number of CCGG sites digested by HpaII or MspI) or the sequential incorporation of A and T nucleotides (reflecting the number of AATT sites digested by EcoRI), were then quantified using the Pyromark Q24 software (Qiagen, France). The level of cytosine methylation was finally determined in duplicate by comparing the ratio of HpaII to MspI cleavage, standardized using EcoRI cleavage.

### Statistical analyses

Results are expressed as median [quartile 1 - quartile 3] and presented as curves (median and interquartile range IQR) or boxplots (minimum to maximum). Statistical analyses were performed using the Rstudio software (version 1.1.447.).

The longitudinal data (mares nutrition, mares weight and BCS, glucose, insulin, urea and amino acid response to diet, growth of foals) were analysed with a mixed linear model using the nlme package^63^, followed with a type 3 Anova (car package^64^). The group (MC, PC or PA), the body weight of the mare, the time and the interaction group:time were taken into account for all the tested variables as fixed effects, the dam was considered as random effect. For metabolic response to diet, gestational age of the mare was included in the model. For foals’ growth, sex of the foal and withers’ height of the mare were included in the model. When group, time, and/or group:time effects were statistically significant, a pairwise post hoc Tukey test was applied using the emmeans package^65^. Placental stereology results were also analysed using a mixed linear model, considering the group (MC, PC or PA), the sex of the foal and the placental sample (umbilical cord, pregnant and non-pregnant horns) as fixed effects and the dam as random effect.

Basal plasma amino acid concentrations before and during the experiment, metabolic tests in mares, placental biometry, ultrastructure, gene expression and methylation results were analysed using a fixed effects linear model, followed with a type 3 Anova (car package^64^) considering the group (MC, PC or PA), the body weight or wither’s height of the mare and the interaction between maternal withers height and analysed variables, and the sex of the foal when statistically significant. When group effect was statistically significant, a pairwise post hoc Tukey test was applied using the emmeans package^65^.

Because sample size was small and statistical power was weak (<50%), p-values from multiple comparisons (metabolic analyses, amino acid concentrations, gene expression) were not corrected. Despite the small sample size, linear models were chosen to correct the data with co-factors that had an effect on the results, such as size of the mares or sex of the foal. Finally, we decided to present statistical tendencies (p-values between 0.05 and 0.10) obtained through Tukey test for comparisons in between groups, only when the group effect was statistically significant (p < 0.05).

The comparison between the amino acid concentrations of mares and foals at birth was analysed using a Multiple Factor Analysis (MFA) on the FactoMineR package^66^. The MFA is a factorial method that enables the study of several groups of variables (in the present paper: dam and foal plasma amino acid concentrations, considering pairs composed of a dam and her foal as a single entity). Maternal results were considered as one group of variables, foal results as the other. Group was considered as qualitative factor. Individuals and variables were graphically represented on the two first dimensions.

Effects were considered significant when p-values < 0.05.

## Supplementary information

Supplementary Figure 1

## Data Availability

All data generated or analysed during this study will be included in this article (as Supplementary Information files) when it will be published.
