# Supplementary material for: Effects of dietary arginine supplementation in pregnant mares on maternal metabolism, placental structure and function and foal growth
Source: Sci Rep. 2019 Apr 23;9:6461. doi: 10.1038/s41598-019-42941-0 (PMC6478728; doi:10.1038/s41598-019-42941-0)

**Effects of dietary arginine supplementation in pregnant mares on maternal metabolism, placental structure and function and foal growth**

Morgane Robles^1^, Anne Couturier-Tarrade^1^, Emilie Derisoud^1^, Audrey Geeverding^1^, Cedric Dubois^2^, Michele Dahirel^1^, Josiane Aioun^1^, Audrey Prezelin^1^, Juliane Calvez^3^, Christophe Richard^1^, Laurence Wimel^2^, Pascale Chavatte-Palmer^1*^

^1^UMR BDR, INRA, ENVA, Université Paris Saclay, Jouy en Josas, France

^2^IFCE, Station Expérimentale de la Valade, Chamberet, France

^3^UMR PNCA, INRA, AgroParisTech, Université Paris Saclay, Paris, France

^*^Correspondence to pascale.chavatte-palmer@inra.fr

**Supplementary Figure 1. Transmission electron microscopy image of placental endothelial cells**

Vesicles (within the cytoplasm) and caveolae (at the basal membrane) of endothelial cells in placentas were counted and quantified per μm2 of cytoplasmic area or length (µm) of basal membrane, respectively.

Most of the vesicles are indicated in blue and caveolins in pink (others are not labeled to allow the reader to see them).

BM: basal membrane; C: caveolae; EC: endothelial cell; FV: fetal vessel; RBC: red blood cell; Ve: vesicle


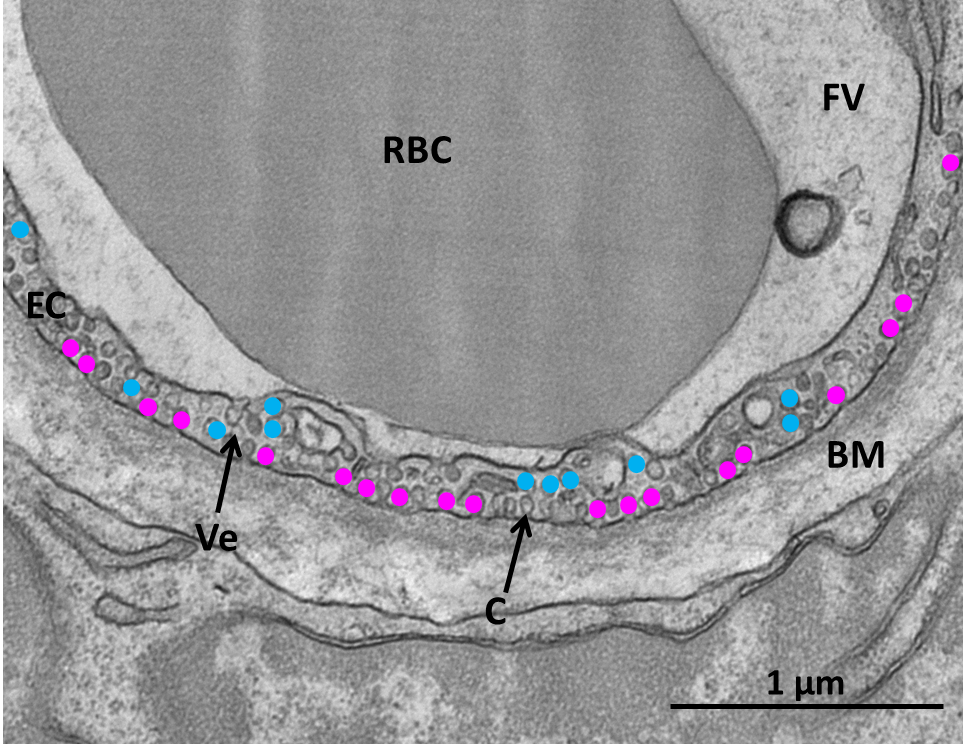

Supplement: Supplementary file 1 — Supplementary Figure 1 [file 41598_2019_42941_MOESM1_ESM.docx]
